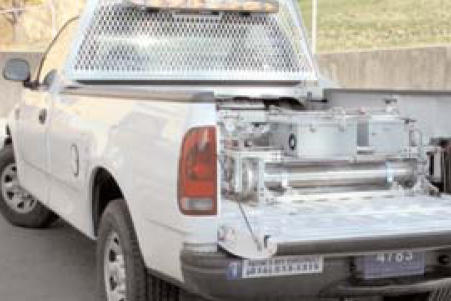# The Beat

**Published:** 2007-07

## Differing Risks from SHS

Research in the March 2007 issue of *Chest* reveals that black children may process secondhand smoke differently than white children do. Black children in the study had blood serum cotinine concentrations that were 32% higher than those of the white children studied. In black children with asthma, hair cotinine levels were up to 4 times higher than in white children with asthma.

This difference could help explain why blacks are more prone to tobacco-related health effects such as cancer, asthma, sudden infant death syndrome, and low birth weight, although the study authors state that more research is required to make definite associations. They add that these findings suggest there also could be differences in exposure or metabolism for other constituents of tobacco smoke, differences that might affect the development of disease.

**Figure:**
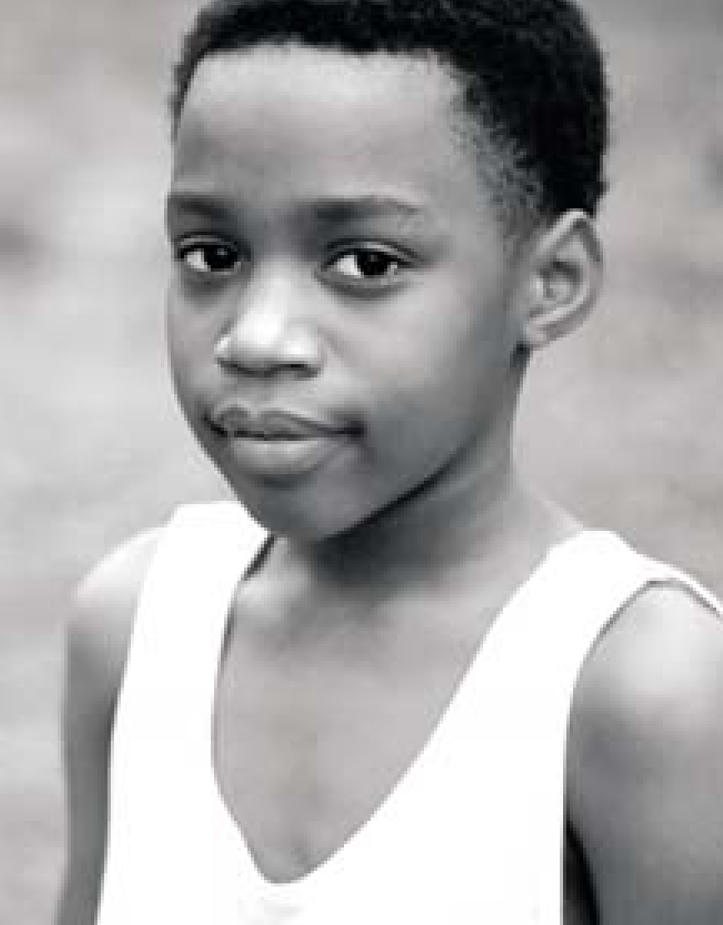


## Rate of Deforestation Slows

A recent UN FAO report contains some good news: the rate of global deforestation has started to slow. Deforestation accounts for 18% of the amount of carbon dioxide produced each year, due to the carbon sequestered in trees being released into the atmosphere. According to *The State of the World’s Forests*, released in March 2007, the implementation of forest replanting projects in some countries has meant that the annual net loss of tree cover has declined from around 9 million hectares a decade ago to 7.3 million now. In addition, between 2000 and 2005, African countries designated 3.5 million hectares of forest as areas for conservation of biological diversity. The FAO found that economic growth can be good for forests, as wealthier countries are more apt to put in place policies for conservation.

## GM Rice Given Go-Ahead

In March 2007 the USDA granted approval for Ventria Bioscience to grow rice engineered to produce human immune proteins. The proteins are intended for use in anti-diarrheal drugs and foods such as yogurt and granola bars. The company says a variety of controls will keep the plants from migrating to surrounding fields or being unintentionally mixed with other grains. Still, science policy and consumer advocacy groups oppose the plan, citing several instances in which genetically modified crops planted outdoors contaminated nearby crops. The same day the USDA released its draft environmental assessment of the Ventria rice project, it also revealed that a separate type of rice had been cross-contaminated with genetically engineered LL62 rice, which has not been released for marketing.

**Figure:**
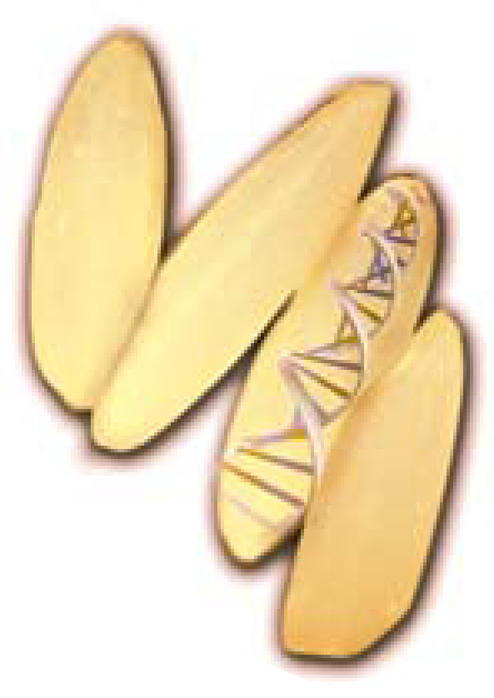


## European Extreme Weather Alert

The weather services of 20 European countries have banded together to form a new website that provides alerts for forecasted weather events such as flash floods, severe thunderstorms, gale-force winds, heat waves, and blizzards, similar to information put out in the United States by the National Weather Service. The website, http://www.meteoalarm.eu/, also supplies 24- and 48-hour warnings for heavy fog, extreme cold, forest fires, and coastal events such as high waves or tides. The Meteoalarm site features a map of Europe with color-coded warnings for each participating country. Clicking on the desired country zooms the visitor in to district-level warnings along with details about the nature of the weather event.

**Figure:**
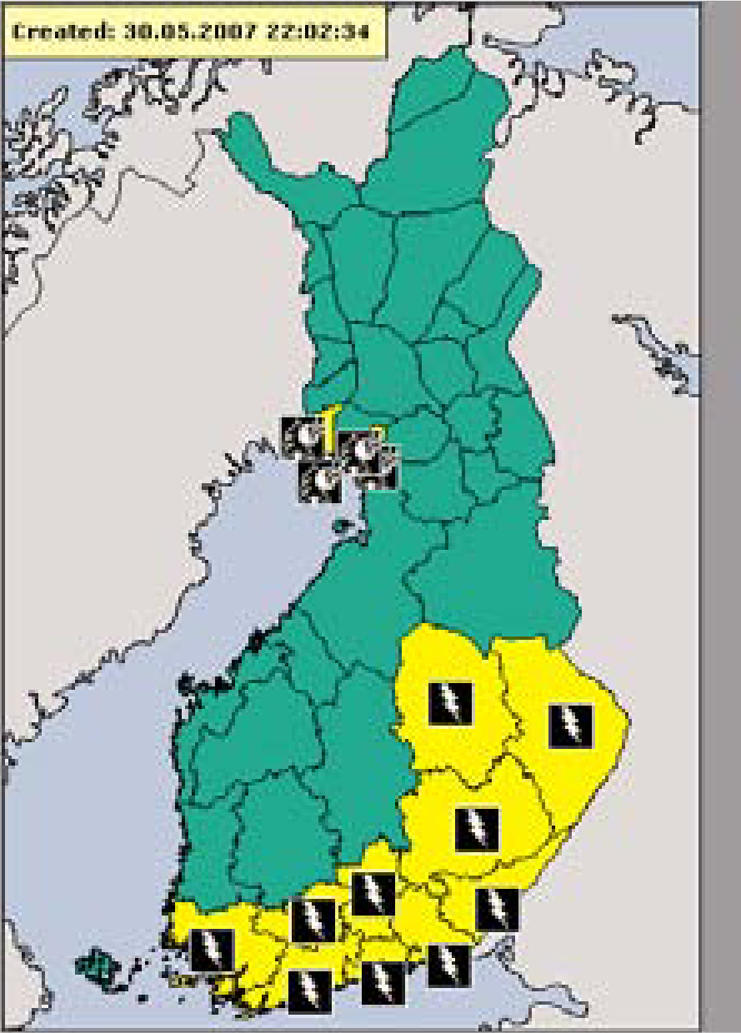


## Continental (Pollution) Drift

Many regions of Australia are in the grip of a drought that has been going on for more than five years. Now, scientists with the Australian research agency CSIRO have found that air pollution in Asia is affecting weather patterns in Australia. The researchers used a new computer climate model that shows aerosol pollution is changing the balance of wind and temperature between Asia and Australia, due to the cooling effect in the atmosphere of the haze of tiny particles from industrial and domestic sources that keeps solar radiation from reaching the Earth’s surface. Currently, the effect is causing more rain in the northwest and center of Australia, and less precipitation in the more populated south and east regions of the continent.

## Solving the Natural Gas Tank Size Puzzle

One of the cleanest burning alternative fuels is natural gas. In light-duty vehicles, natural gas produces 90% less carbon monoxide, 60% less nitrogen oxides and between 30 and 40% less carbon dioxide than gasoline. Until now, the use of natural gas in vehicles has been limited by the fact that the fuel storage tanks necessary were very bulky and required high-pressure conditions. Researchers at the University of Missouri–Columbia and Midwest Research Institute have come up with a solution in which ground corncobs are baked and compressed into carbon briquettes. These briquettes contain “fractal pore spaces”—spaces created by repetition of similar patterns at different levels of magnification—that can store 180 times their own volume of natural gas without high pressure and that can be fit into smaller areas. The technology is currently being tested in a truck owned and operated by the Kansas City Office of Environmental Quality.

**Figure:**